# Comparison of maintenance and emergence characteristics after desflurane or sevoflurane in outpatient anaesthesia

**DOI:** 10.4103/0019-5049.76604

**Published:** 2011

**Authors:** Ravi Jindal, Ved Prakash Kumra, Krishan Kumar Narani, Jayashree Sood

**Affiliations:** Department of Anaesthesiology, Pain and Perioperative Medicine, Sir Ganga Ram Hospital, New Delhi, India

**Keywords:** Desflurane, outpatient, recovery, sevoflurane

## Abstract

Both sevoflurane and desflurane have shorter emergence times compared to isoflurane based anaesthesia. Because of its pharmacological properties, desflurane appears to yield a rapid early and intermediate recovery compared with sevoflurane. The aim of this study was to assess the maintenance and emergence characteristics after anaesthesia with sevoflurane or desflurane. One hundred female patients scheduled to undergo daycare laparoscopic gynaecological surgery were enrolled for this prospective study. Patients were randomised into two groups to receive either desflurane [group I (D); n = 50] or sevoflurane [group II (S); n = 50] for maintenance of anaesthesia. The demographic data and the duration of procedure were comparable in both the groups. The early recovery time was shorter after maintenance of anaesthesia with desflurane compared with sevoflurane. However, this faster early recovery failed to lead to early readiness for home discharge. The intraoperative haemodynamic characteristics were comparable with both sevoflurane and desflurane. Both sevoflurane and desflurane provide a similar time to home readiness despite a faster early recovery with desflurane. The intraoperative haemodynamics are similar with both the agents.

## INTRODUCTION

“Ambulatory anaesthesia” is as old as “anaesthesia” itself and now contributes to a greater proportion of overall surgeries than it did 10 years ago. This is because of the availability of improved minimally invasive surgical techniques and addition of new short acting and rapidly metabolising anaesthetic agents. Very high-risk patients and major surgical procedures can now be conducted safely because of the precision in monitoring and advanced surgical techniques.[1]

One of the major factors that determine speed of recovery from anaesthesia is the choice of anaesthetic technique. An ideal general anaesthetic, for the ambulatory patients, should provide smooth and rapid induction, optimal operating conditions, and rapid recovery with minimal side effects like nausea, vomiting, bleeding and postoperative pain.

Inhaled anaesthetics allow rapid emergence from anaesthesia because of easy titrability with inherent neuromuscular blocking[2] effects that make them more suitable for ambulatory anaesthesia. The availability of less soluble inhalation anaesthetics such as sevoflurane and desflurane made us rethink about the selection of volatile anaesthetics for outpatient surgical procedures. Given the low blood: gas partition coefficient of sevoflurane and desflurane, faster emergence from anaesthesia is expected compared to traditional inhalation anaesthetics.[3]

Sevoflurane, a volatile anaesthetic agent, is halogenated ether. It has rapid induction due to low blood: gas partition (blood: gas partition coefficient of 0.65 and fat: blood solubility 48 at 37°C). Desflurane is also halogenated ether. Low solubility of desflurane in blood and body tissues (blood: gas partition coefficient of 0.42 and fat: blood solubility 27 at 37°C) leads to rapid induction and recovery.[4,5]

Both sevoflurane and desflurane have shorter emergence times compared to isoflurane-based anaesthesia techniques.[3] Because of its pharmacological properties, desflurane appears to yield a rapid early and intermediate recovery compared with sevoflurane. However, the results of different studies have been conflicting. Also, desflurane has only recently become available in India and has yet not been studied for daycare laparoscopic surgery in Indian population.

The purpose of this prospective randomised study was to assess the maintenance and emergence characteristics after anaesthesia with sevoflurane or desflurane for outpatient gynaecological laparoscopic surgeries. The aim was to analyse and compare the superiority of each agent, with regards to faster emergence, early and intermediate recovery. The intraoperative haemodynamic profile and postoperative side effects of the two agents were also analysed.

## METHODS

One hundred female patients belonging to ASA grade I or II, scheduled to undergo short daycare gynaecological procedures (lasting between 30 and 90 min) were recruited for this randomised, prospective, single-blind study. Hospital ethics committee approval and a written informed consent from all patients were taken. The patients were randomised into two groups to receive either desflurane [group I (D); *n* = 50] or sevoflurane [group II (S); *n* = 50] for maintenance of anaesthesia.

A pre-anaesthetic examination comprising history, general physical and systemic examination of all the patients was conducted. Routine investigations including haemoglobin, total leucocyte count, blood sugar, serum creatinine and urine examination were carried out. All patients were kept fasting for at least 6 hours prior to surgery.

The study excluded the patients with significant cardiopulmonary disease, hepatic or renal dysfunction, endocrinal disturbances, neurological or psychiatric disorder, those with history of drug allergy or drug abuse, those on central nervous system (CNS) depressants, pregnant/breastfeeding females and those who had undergone recent anaesthesia (within the previous 7 days).

Patients were randomly divided into two groups of 50 each by a computer generated randomisation sheet as follows:

Group I (D) – anaesthesia was induced using propofol and maintained with 60% N_2_O in O_2_and desflurane and

Group II (S) – anaesthesia was induced using propofol and maintained with 60% N_2_O in O_2_and sevoflurane.

Tab. Alprazolam 0.25 mg and Tab. Pantoprazole 40 mg were given as premedication 60 min prior to induction to all the patients.

In the operating room, an intravenous (IV) line was secured on the non-dominant hand of the patient, monitors were attached and baseline heart rate (HR), mean arterial pressure (MAP) and oxygen saturation (SpO_2_) were recorded.

All patients received fentanyl citrate 2 mcg/kg intravenously and were preoxygenated prior to induction of anaesthesia. Anaesthesia was induced with propofol 1.5 mg/kg IV. After loss of consciousness, ventilation of lungs was manually assisted. Neuromuscular blockade was achieved with vecuronium bromide 0.1 mg/kg IV and airway secured with an endotracheal tube.

The patients subsequently received either sevoflurane 1–2% or desflurane 3–6% with 60% nitrous oxide in oxygen. The inspired concentration of the volatile anaesthetic was adjusted to maintain MAP within 20% of baseline values. Additional rescue bolus doses of fentanyl citrate 0.5-0.75 mcg/kg were administered to control acute haemodynamic changes not responding to a 50% increase in inspired concentration of the volatile drug.

Muscle relaxation was maintained using intermittent doses of vecuronium bromide at appropriate intervals.

Monitoring was done using SpO_2_, non-invasive blood pressure (NIBP), electrocardiogram (ECG), HR and end-tidal carbon dioxide (EtCO_2_). The maintenance dose of anaesthetics was titrated to maintain a bispectral index (BIS) value of 40–60. All the patients were ventilated to maintain an EtCO_2_of 32–36 mm Hg.

The primary anaesthetic was discontinued at the end of the procedure and N_2_O was discontinued after the last skin suture was placed. The neuromuscular block was reversed with Inj. glycopyrrolate 0.008 mg/kg and Inj. neostigmine 0.05 mg/kg intravenously.

In the post anaesthesia care unit (PACU), all the patients were nursed in propped up position. Oxygen was administered via Hudson mask and the recovery characteristics were recorded every 5 min with the help of Modified Aldrete Scoring System. Discharge from PACU was decided once the score was 9 or above.

Post Anaesthetic Discharge Scoring System (PADSS) was used to assess time to home readiness at an interval of 15 min. Score of 9 and above made the patient fit to go home.

Patients were observed for nausea / vomiting, drowsiness, respiratory distress and pain postoperatively and treated with ondansetron hydrochloride dihydrate 0.1 mg/kg body weight IV, nebulisation with salbutamol sulphate 2.5 mg in 2.5 ml normal saline or fentanyl citrate 0.5 mg/kg body weight IV in case of these complications.

The recovery patterns with different anaesthetic techniques were analysed statistically.

A study population of 50 patients for each group was determined to have 99% power at α = 0.05 (two-tailed) to detect a difference of 10% in the time to early recovery with desflurane group compared to sevoflurane group.

Data were expressed as mean ± SD. Statistical analysis of data among the groups was done by Student’s *t* test for independent samples, and for categorical value, Fisher’s exact test was applied. For non-parametric data, Mann-Whitney U test was used.

*P* value <0.05 was considered as statistically significant. SPSS 17 statistics package was used for analysis.

## RESULTS

One hundred patients were recruited for the study. Fifty patients were allocated in each group. There was no premature study withdrawal due to failure of surgery to proceed as planned or the development of complications hindering the assessment of study variables. Patient characteristics as well as duration of anaesthesia and surgery were comparable in both the groups [Table 1].

**Table 1:** Demographic and other data

	Group I (D)	Group II (S)	*P* value
Age (years)	33.2 (7.290)	30.36 (4.219)	0.064 (NS)
Weight (kg)	58.72 (7.360)	57.20 (9.012)	0.358 (NS)
Height (cm)	154.58 (2.997)	155.19 (2.665)	0.284 (NS)
Duration of anaesthesia (min)	51.30 (9.784)	53.16 (12.016)	0.398 (NS)
Duration of procedure (min)	38.9 (8.765)	41.3 (11.641)	0.247 (NS)

Values are expressed as mean (SD); *P* < 0.05 is significant; NS, not significant

The patients were anaesthetised for a mean duration of 51.30 and 53.16 min in desflurane and sevoflurane groups, respectively, the differences being statistically insignificant [Table 1]. The mean duration of surgery in the groups of desflurane and sevoflurane was 38.90 and 41.30 min, respectively, with no statistical difference between them [Table 1].

**Figure 1:**
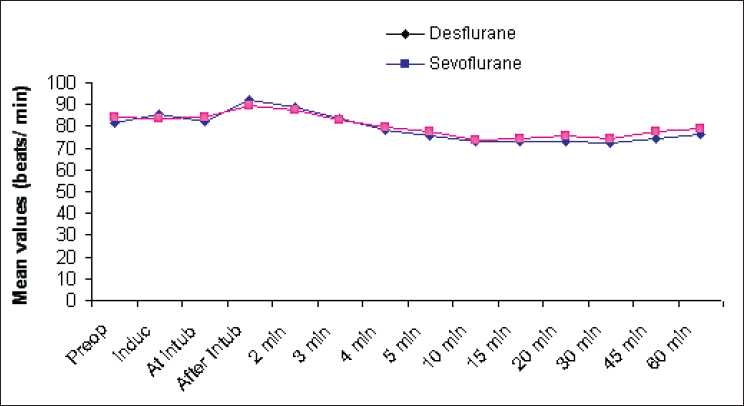
Comparison of heart rate

**Figure 2:**
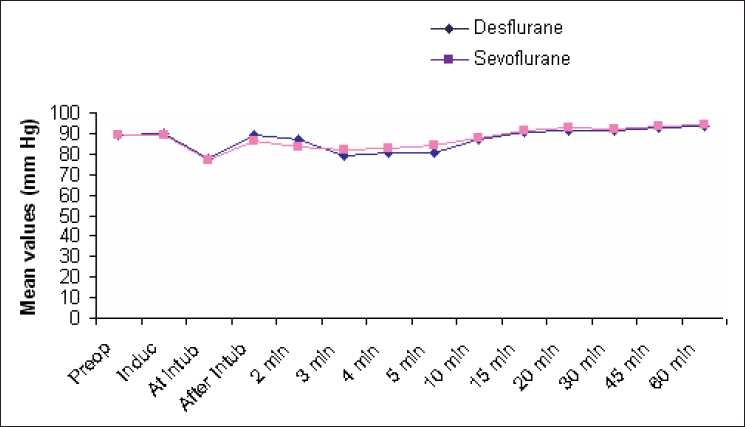
Comparison of mean arterial pressure

There was no statistical difference in the intraoperative HR and mean arterial blood pressure between the groups [Tables 2 and 3, Figures 1 and 2].

**Table 2:** Heart rate (beats/min)

	Group I (D)	Group II (S)	*P* value
Preoperative	81.58 (10.025)	83.98 (10.790)	0.252 (NS)
Induction	85.32 (12.891)	83.54 (10.752)	0.455 (NS)
At intubation	82.36 (12.116)	84.34 (13.514)	0.442 (NS)
Immediately after intubation	92.02 (12.290)	89.26 (13.521)	0.288 (NS)
2 min	88.96 (11.150)	87.32 (13.942)	0.517 (NS)
3 min	83.64 (10.883)	82.94 (11.883)	0.759 (NS)
4 min	78.54 (11.433)	79.70 (11.182)	0.609 (NS)
5 min	75.56 (11.207)	77.44 (11.418)	0.408 (NS)
10 min	73.00 (11.053)	73.58 (11.720)	0.800 (NS)
15 min	73.08 (11.130)	74.42 (11.009)	0.546 (NS)
20 min	72.90 (10.403)	75.72 (10.958)	0.190 (NS)
30 min	72.16 (10.001)	74.38 (8.051)	0.224 (NS)
45 min	74.54 (9.671)	77.38 (9.005)	0.132 (NS)
60 min	76.24 (7.968)	79.14 (8.804)	0.087 (NS)

Values are expressed as mean (SD); *P* < 0.05 is significant; NS, not significant

**Table 3:** Mean arterial pressure (mm Hg)

	Group I (D)	Group II (S)	*P* value
Preoperative	89.013 (9.371)	89.160 (7.891)	0.933 (NS)
Induction	89.98 (8.823)	89.31 (10.933)	0.723 (NS)
At intubation	78.01 (12.111)	76.65 (9.874)	0.538 (NS)
Immediately after intubation	89.43 (11.083)	86.59 (11.576)	0.189 (NS)
2 min	86.85 (11.723)	83.71 (10.404)	0.160 (NS)
3 min	79.007 (10.220)	82.16 (11.175)	0.144 (NS)
4 min	80.56 (9.822)	82.68 (13.411)	0.369 (NS)
5 min	80.360 (9.406)	84.333 (11.605)	0.063 (NS)
10 min	90.82 (7.482)	91.26 (10.515)	0.810 (NS)
15 min	91.56 (7.941)	92.953 (9.791)	0.436 (NS)
20 min	89.733 (7.770)	92.953 (9.791)	0.072 (NS)
30 min	91.307 (9.670)	91.767 (12.023)	0.833 (NS)
45 min	92.47 (7.609)	93.80 (8.549)	0.414 (NS)
60 min	93.63 (5.974)	94.09 (8.596)	0.757 (NS)

Values are expressed as mean (SD); *P* < 0.05 is significant; NS, not significant

The time from administration of reversal agent to response to painful stimuli, to eye opening, to verbal commands and spontaneous eye opening were significantly shorter in patients administered desflurane than in patients given sevoflurane. For a given duration of anaesthesia, emergence from anaesthesia was significantly faster in desflurane compared to sevoflurane group [Table 4].

Patients given desflurane achieved Modified Aldrete Score of 9 significantly faster than patients given sevoflurane [Table 4]. The two groups were comparable with respect to time to achieve PADSS of 9 or above, the difference being insignificant [Table 4]. There was no difference in both the groups as far as the incidence of postoperative complications was concerned [Table 5].

**Table 4:** Recovery (min)

		Group I (D)	Group II (S)	*P* value
Response to painful stimuli		2.75 (1.411)	4.02 (1.767)	0.000 (S)
Response to verbal commands		3.48 (1.488)	5.04 (1.616)	0.000 (S)
Spontaneous eye opening		4.18 (1.548)	6.80 (2.259)	0.000 (S)
Stating name		5.34 (1.944)	7.62 (2.079)	0.000 (S)
Date of birth		5.56 (1.955)	8.00 (2.399)	0.000 (S)
Place of stay		5.66 (1.955)	8.16 (2.289)	0.000 (S)
Squeeze fingers		6.76 (2.016)	9.36 (2.038)	0.000 (S)
Lift limb		7.14 (2.250)	10.08 (1.947)	0.000 (S)
Modified aldrete score	Arrival	8.42 (0.785)	8.02 (0.622)	0.006 (S)
	After 5 min	9.36 (0.598)	9.00 (0.808)	0.013 (S)
	After 10 min	9.96 (0.198)	9.88 (0.385)	0.195 (NS)
Time to achieve modified aldrete score of 9 Readiness to home discharge		10.80 (3.774)	16.20 (3.870)	0.000 (S)
Time to achieve PADSS of 9 or above (min)		188.40 (22.302)	193.20 (22.605)	0.288 (NS)

Values are expressed as mean (SD); *P* < 0.05 is significant; S: Significant; NS: Not significant; PADSS: Post anaesthetic discharge scoring system

**Table 5:** Postoperative complications

	Group I (D)	Group II (S)	*P* value
Nausea and vomiting	35/50	38/50	0.499 (NS)
Drowsiness	4/50	3/50	0.773 (NS)
Respiratory distress and laryngospasm	6/50	5/50	0.749 (NS)
Sore throat	2/50	1/50	0.556 (NS)
Headache	0/50	0/50	—

Values are expressed as mean (SD); *P* < 0.05 is significant; NS: not significant

## DISCUSSION

Emergence and early recovery from anaesthesia was faster with desflurane compared to sevoflurane. However, the intermediate recovery end points (readiness for home discharge) did not differ significantly between the two anaesthetic groups. As a result of the lower solubility of desflurane compared with sevoflurane in blood and lean tissues, one might expect to find differences in the intermediate and late recovery end points when these two anaesthetics are used for longer surgical procedures. However, results of studies have been conflicting.

The results of this study do not fully support the earlier study by Mahmoud, reporting that the faster emergence after discontinuation of desflurane led to an earlier discharge and more rapid resumption of normal activities compared with sevoflurane.[6] Similarly, Karlsen found no significant differences between desflurane and sevoflurane during recovery.[7]

The current findings are consistent with previously published comparative studies demonstrating that the faster emergence from desflurane (versus sevoflurane) anaesthesia failed to lead to an earlier discharge from hospital after both outpatient and inpatient surgical procedures.[8–16]

The study by Nathanson suggested that sevoflurane and desflurane provided similar intraoperative conditions during the maintenance period. Although early recovery was faster with desflurane, there was no difference in the intermediate recovery end points.[8] Song also found that late recovery profiles and incidence of postoperative side effects were similar after desflurane and sevoflurane administration.[9] White concluded that despite the faster initial recovery with desflurane, no significant differences were found between the two volatile anaesthetics in the later recovery period.[10] Isik and others also concluded that in children, early recovery was faster with desflurane compared to sevoflurane.[11] Findings of the present study are consistent with the earlier reported data of faster early recovery with desflurane compared to sevoflurane. This is despite the use of BIS as an additional monitor (which was not used in most of the earlier studies) to titrate maintenance anaesthetic, a lower induction and maintenance dose of other anaesthetic agents compared with earlier studies and South Asian population instead of western population.

Eger in a study found that for a given duration of anaesthesia, elimination was faster and recovery was quicker for desflurane.[12] Other studies[8,13–16] have found that only early recovery was faster with desflurane compared to sevoflurane even when the duration of surgery exceeded 2 hours. Furthermore, the recovery of psychomotor and cognitive function after desflurane and sevoflurane administration were similar after the first 30–45 min in both younger patients undergoing ambulatory surgery and elderly patients undergoing more prolonged general anaesthesia for inpatient procedures. Yet, even these studies accept the possibility that the difference in intermediate recovery and cognitive function recovery might not have been detected due to lack of sensitivity and selectivity of “digit-symbol substitution test” and “mini-mental state test” used in these studies.

Intraoperative cardiovascular stability was easily achieved with both sevoflurane and desflurane, with MAP and HR maintained within ±20% of baseline values during the entire maintenance period. Further, the use of BIS monitoring helped standardise the depth of anaesthesia and maintain the same in a consistent manner relative to many other studies.

Gergin studied the haemodynamics, emergence and recovery characteristics of sevoflurane with those of desflurane in nitrous oxide anaesthesia and concluded that the groups did not differ in these haemodynamic measures.[17] However, a study by Elbert concluded that neurocirculatory excitation seen with rapid increase in desflurane did not occur with sevoflurane. At steady state, increasing the concentration of sevoflurane was associated with lower sympathetic nerve activity and central venous pressure.[18] Our study supported the findings in the former group.

In our study, we found that both desflurane and sevoflurane groups had rapid recovery. There was a significant difference in the emergence and early recovery between the two groups. The early recovery was faster with desflurane compared to sevoflurane. Although there was difference in intermediate recovery time, the magnitude of the difference was small and insignificant. This was despite not allowing stepwise reduction of anaesthetic concentration towards the end of procedure, which is a common clinical practice. One explanation for this small magnitude of difference could be the residual effects of drugs used for premedication, opiates and muscle relaxants, which could have interfaced with the anaesthetic agents. Short duration of anaesthesia might also have masked any difference in intermediate and late recovery.

Because of the greater pungency and airway irritant properties of desflurane, sevoflurane has been called “the ideal agent for adult day case surgeries anaesthesia”. Previous studies comparing desflurane and sevoflurane found that sevoflurane causes moderate bronchodilatation not observed with desflurane[19] and sevoflurane was significantly less irritating to airways compared to desflurane.[20,21] Other studies comparing desflurane and sevoflurane when administered for minor outpatient surgical procedures using a laryngeal mask airway for airway management have reported a low incidence of respiratory complications and no significant differences between the two volatile anaesthetics.[22–24] Our study found no difference in the incidence of respiratory complications between the two groups, which could be attributed to propofol used for induction. It is also possible that the use of fentanyl during the intraoperative period may have minimised the difference between the airway responses to desflurane and sevoflurane.

The incidence of other postoperative complications (postoperative nausea and vomiting, headache, drowsiness) was also similar in both the groups. This was in contrast to a study by Karlsen who found that the postoperative nausea/vomiting rate (24 hour in PACU and ward) was higher in the desflurane group (67%) than that in sevoflurane group (36%).[7]

This study can be criticised because the design did not permit a double-blind comparison of the two volatile anaesthetics. However, all patients were undergoing identical procedure and the anaesthesiologists maintained BIS values in the range of 40–60.

In addition, a cost analysis with the use of the two agents was not a part of this study. The cost saving due to lesser use of opioids, muscle relaxants, carrier gases, and early discharge from the PACU could impinge on the decision regarding use of either sevoflurane or desflurane in an ambulatory setting.

## CONCLUSION

The emergence and early recovery time were shorter after maintenance of anaesthesia with desflurane compared with that of sevoflurane. Hence, wake up time was less with desflurane than with sevoflurane. However, this faster wake up failed to translate into early readiness for home discharge. The intraoperative haemodynamic characteristics were comparable with both sevoflurane and desflurane. The incidence of postoperative complications was also similar in both the groups.

Thus, it is concluded that both sevoflurane and desflurane provide a similar time to home readiness despite a faster wake up time with desflurane. The intraoperative haemodynamics is similar with both the agents.

## References

[1] Gupta A, Stierer T, Zuckerman R, Sakima N, Parker SD, Fleisher LA (2004). Comparison of recovery profile after ambulatory anesthesia with propofol, isoflurane, sevoflurane and desflurane: A systematic review. Anesth Analg.

[2] Eriksson LI (1999). The effects of residual neuromuscular blockade and volatile anesthetics on control of ventilation. Anesth Analg.

[3] Nathanson MH, Fredman B, Smith I, White PF (1995). Sevoflurane versus desflurane for outpatient anaesthesia: A comparison of maintenance and recovery profiles. Anesth Analg.

[4] Eberts TJ, Schmid PG, Barash PG, Cullen BF, Stoelting RK, Cahalan MK, Stock MC (2009). Inhaled anesthetics. Clinical Anesthesia.

[5] Morgan GE, Mikhail MS, Murray MJ (2006). Inhalational anesthetics. Clinical Anesthesiology.

[6] Sjosvard NK, Sjoberg F, Gupta A (1998). Anaesthesia for videoarthroscopy of the knee. A comparison between desflurane and sevoflurane. Acta Anaesth Scand.

[7] Karlsen KL, Persson E, Wennberg E (2000). Anaesthesia, recovery and postoperative nausea and vomiting after breast surgery. A comparison between desflurane, sevoflurane and isoflurane anaesthesia. Acta Anaesth Scand.

[8] Dupont J, Tavernier B, Ghosez Y, Durinck L, Thevenot A, Moktadir-Chalons N (1999). Recovery after anaesthesia for pulmonary surgery: Desflurane, sevoflurane and isoflurane. Br J Anaesth.

[9] Welborn LG, Hannallah RS, Norden JM, Ruttimann UE, Callan CM (1996). Comparison of emergence and recovery characteristics of sevoflurane, desflurane and halothane in pediatric ambulatory patients. Anesth Analg.

[10] Mahmoud NA, Rose JA, Laurence AS (2001). Desflurane or sevoflurane for gynaecological day case anaesthesia with spontaneous respiration. Anaesthesia.

[11] Isik Y, Goksu S, Kocoglu H, Oner U (2006). Low flow desflurane and sevoflurane anaesthesia in children. Eur J Anaesthesiol.

[12] Eger EI, Gong D, Koblin DD, Bowland T, Ionescu P, Laster MJ (1998). The effect of anesthetic duration on kinetic and recovery characteristics of desflurane versus sevoflurane, and on the kinetic characteristics of compound A, in volunteers. Anesth Analg.

[13] Juvin P, Servin F, Giraud O, Desmonts JM (1997). Emergence of elderly patients from prolonged desflurane, isoflurane, or propofol anaesthesia. Anesth Analg.

[14] Heavner JE, Kaye AD, Lin BK, King T (2003). Recovery of elderly patients from two or more hours of desflurane or sevoflurane anaesthesia. Br J Anaesth.

[15] Rörtgen D, Kloos J, Fries M, Grottke O, Rex S, Rossaint R (2010). Comparison of early cognitive function and recovery after desflurane or sevoflurane anaesthesia in the elderly: A double-blinded randomized controlled trial. Br J Anaesth.

[16] Dexter F, Bayman EO, Epstein RH (2010). Statistical modeling of average and variability of time to extubation for meta-analysis comparing desflurane to sevoflurane. Anesth Analg.

[17] Bedforth NM, Hardman JG, Nathanson MH (2000). Cerebral haemodynamic response to the introduction of desflurane: A comparison with sevoflurane. Anesth Analg.

[18] Ebert TJ, Muzi M, Lopatka CW (1995). Neurocirculatory responses to sevoflurane in humans: A comparison to desflurane. Anesthesiology.

[19] Goff MJ, Arain SR, Ficke DJ, Uhrich TD, Ebert TJ (2000). Absence of bronchodilation during desflurane anesthesia-A comparison to sevoflurane and thiopental. Anesthesiology.

[20] TerRiet MF, DeSouza GJ, Jacobs JS, Young D, Lewis MC, Herrington C (2000). Which is most pungent: Isoflurane, sevoflurane or desflurane?. Br J Anaesth.

[21] Klock PA, Czeslick EG, Klafta JM, Ovassapian A, Moss J (2001). The effect of sevoflurane or desflurane on upper airway reactivity. Anesthesiology.

[22] Eshima RW, Maurer A, King T, Lin BK, Heavner JE, Bogetz MS (2003). A comparison of airway responses during desflurane and sevoflurane administration via a laryngeal mask airway for maintenance anesthesia. Anesth Analg.

[23] McKay RE, Large MJ, Balea MC, McKay WR (2005). Airway reflexes return more rapidly after desflurane anesthesia than after sevoflurane anesthesia. Anesth Analg.

[24] McKay RE, Bostrom A, Balea MC, McKay WR (2006). Airway responses during desflurane versus sevoflurane administration via a laryngeal mask airway in smokers. Anesth Analg.

